# The Effects of Trauma, Violence, and Stress on Sexual Health Outcomes Among Female Clinic Clients in a Small Northeastern U.S. Urban Center

**DOI:** 10.1089/whr.2019.0027

**Published:** 2020-05-19

**Authors:** Natalie M. Leblanc, Kamila Alexander, Sierra Carter, Hugh Crean, Ladrea Ingram, James Kobie, James McMahon

**Affiliations:** ^1^ School of Nursing, University of Rochester Medical Center, Rochester, New York, USA.; ^2^ Department of Community Public Health Nursing, Johns Hopkins University School of Nursing, Baltimore, Maryland, USA.; ^3^ Department of Psychology, Georgia State University, Atlanta, Georgia, USA.; ^4^ Atrium Health, Charlotte, North Carolina, USA.; ^5^ Department of Medicine, University of Alabama at Birmingham, Birmingham, Alabama, USA.

**Keywords:** health disparities in sexual health, sexual behavior, women's sexual health

## Abstract

***Background:*** Threats to sexual health can emerge across one's life span and are influenced by individual and interpersonal experiences, as well as certain environmental exposures. Although previous research has recognized the importance of ecological frameworks to understand the complexity of health and behaviors in marginalized communities, there continues to be a dearth of research that truly utilizes this perspective to gain insight into the multifaceted factors that can concurrently influence threats to sexual health among women.

***Methods:*** A sample of 279 ethnoracially diverse women were recruited from a U.S. northeastern small urban center health clinic to participate in a parent study on trauma and immunity. A hierarchical block analysis was conducted to investigate associations between women's experiences of trauma, stress and violence (*i.e.*, childhood trauma (CHT), intimate partner violence (IPV), neighborhood stressors), and sexual health outcomes and behaviors (*i.e.*, lifetime sexually transmitted infection [STI] diagnosis, concurrent partnerships, and lifetime sex trading).

***Results:*** In the full hierarchical model, IPV and life stress trauma were associated with lifetime sex trading and partner concurrency. Also in the full model, sexual CHT was associated with lifetime STI acquisition and partner concurrency, while emotional CHT was associated with lifetime sex trading. Lastly, as neighborhood disorder increased, so did the number of lifetime sex trading partners.

***Conclusion:*** Sexual health assessments in clinical and community settings require a holistic, comprehensive, and meaningful approach to inform person-centered health promotion intervention. Prevention and treatment interventions require a focus on parents and families, and should assist adolescents and young adults to adopt therapies for healing from these experiences of trauma, violence, and stress. Interventions to enhance sexual health promotion must also include the following: advocacy for safe environments, social policy that addresses lifelong impacts of CHT, and fiscal policy that addresses economic vulnerability among women and threatens sexual health. Further recommendations are discussed.

## Introduction

Experiences of trauma and stress across the life span influence the development of adverse health outcomes and engagement in behaviors that threaten health and wellness.^1–3^ Sexual health outcomes in the context of traumatic and stressful experiences are of particular interest due to the (1) interpersonal transmission of sexually transmitted infections (STIs); (2) lifelong health impact of STIs when untreated or are recurrent (*i.e.*, infertility, pelvic inflammatory disease) or those which are incurable (*i.e.*, HPV and HIV); and (3) established associations between violence, trauma, and stress as threats to sexual health.^4^

Sexual health involves a sex-positive perspective on sexuality, sexual relationships, and sexual behaviors that incorporates experiences free of coercion and violence, and prevention and/or treatment of diseases transmitted sexually.^5^ Threats to sexual health can involve individual behaviors, interpersonal and ecological factors that heighten one's exposure to acquiring or transmitting an STI, or a context that compromises volitional control over one's sexuality, sexual behavior, and sexual safety.^5–7^

The current study is a secondary data analysis that investigates threats to sexual health among women recruited from health clinic in a small US northeastern urban center. Although previous research has recognized the importance of ecological frameworks to understand the complexity of health and health behaviors in marginalized communities, there continues to be a dearth of research that truly utilizes this perspective to gain insight into the multifaceted factors that can concurrently influence threats to sexual health among women. This analysis uses hierarchical modeling to investigate multilevel ecological influences across the life span: (1) interpersonal violence (sexual, emotional, and physical childhood trauma [CHT]; lifetime intimate partner violence [IPV]), and (2) stressors (life stress and environmental stress [city violence and neighborhood disorder]) on sexual health and behavioral outcomes. The current study investigates the relationship of these experiences with three specific sexual health outcomes and behaviors: lifetime history of STIs, current partner concurrency (defined as sexual partners overlapping in time), and lifetime sex trading (defined as exchanging sexual activity for food, drugs, or money). These three outcomes have been associated with one another, recurrent HIV/STI transmission, other nonsex-based adverse health outcomes, and contextual behaviors that have implications for one's sexual health and autonomy.^6^

A history of STIs among women is not only indicative of unprotected sex acts,^7^ but in some contexts has also been associated with early childhood sexual trauma, IPV as an adult, and community/society-level detriments.^8–11^ One systematic review found relationships between increased vulnerability to HIV/STI acquisition *via* decreased condom and oral contraceptive use and women experiencing IPV.^12^ Haley et al.^11^ found that among 737 U.S. women, an STI diagnosis was associated with neighborhood social disorder and social disadvantage. A South African longitudinal study among a sample of young women found that HIV incidence was highest among those who engaged in greater frequency (weekly) of transactional sex for goods and/or money.^13^ A U.S. study among a sample of 346 women demonstrated that those who reported sex trading (28%) were more likely to experience IPV, drug use, and homelessness.^14^ These findings implicate the need to consider the broader social environment when investigating influences on sexual health outcomes. Given the multifaceted influences on and relationships among adverse sexual health outcomes and behaviors, we hypothesized that the experiential impact of interpersonal violence and stressors occurring at multiple levels across one's life span will be associated with sexual behaviors and outcomes. Although research has demonstrated links between HIV/STI transmission, and social and individual-level factors such as drug and condom use, few have examined unique connections between individual, interpersonal, and environmental experiences on HIV/STI-associated behaviors and sexual health outcomes concurrently.^15,16^ Contextualizing these factors and experiences is essential for promoting comprehensive sexual health and wellness strategies that address outcomes across the life span and are inclusive of HIV/STI prevention and treatment interventions and policies.

## Materials and Methods

### Participants

Participants (*N* = 279) were recruited in 2015 from a publicly funded sexual health clinic in a small urban center in the northeastern United States. Participants were enrolled in a parent research project about life traumatic experiences and immunologic outcomes (BLINDED IRB #55934). All participants were female who reported either being in a primary relationship with a main male partner and/or reported male sexual partners within the past 3 months. Participants ranged from 19 to 64 years of age (mean age = 28 years). The sample was 65% African American, 18.6% white American, and comprised other groups that included multiracial/mixed (9%), Asian (4%), Native American (1.4%), and self-identified ethnoracial identities (3.6%; *i.e.*, Italian, Eritrean, Hispanic/Latinas). Approximately 50% of the women had a high school education or less, 64% were employed, and 83% earned less than $30,000 a year. Many participants reported being in a primary steady relationship (41%) for 1 year or more. Additional demographic details are given in Tables 1 and 2.

**Table 1.** Demographics

Variable/construct	N	Proportion/range
Age	279	18–64
Race	279	
African American/Black	183	65.6%
White/Caucasian	52	18.6%
Other	44	
Marital status	279	
Ever married	31	11.1%
Single	248	88.9%
Employment	279	
Full-time	111	39.8%
Part-time	68	24.4%
Unemployed	100	35.8%
Education	279	
High school or less	132	47.3%
Some college	102	36.6%
College graduate	45	16.1%
Family income	277	
≤$15,000	138	49.5%
$15,000−$30,000	94	33.7%
≥$30,000	45	16.2%

**Table 2.** Sample Characteristics

Selected constructs (N)	Responses	Response (%)	Range (mean)
Childhood emotional trauma			
	Yes	261 (93%)	13–62 (32)
	No	18	
Childhood physical trauma			
	Yes	224 (80%)	8–42 (15)
	No	55	
Childhood sexual trauma			
	Yes	100 (36%)	3–25 (8.2)
	No	179	
IPV			
	Yes	67 (24%)	3–18 (5.7)
	No	212	
Life stress			
	Yes	279	1–150 (47.5)
	No	0	
City violence exposure			
	Yes	202 (72%)	0–18 (3)
	No	77	
Neighborhood disorder			
	Yes	259 (96%)	0–36 (15.6)
	No	10	
Lifetime STI			
	≤1	115 (42%)	
	2–3	81 (30%)	
	≥4	76 (28)	
Partner concurrency			
	Yes	79 (28%)	
	No	200	
Lifetime sex trading			
	Yes	51 (19%)	
	No	218	

IPV, intimate partner violence; STI, sexually transmitted infection.

### Data management and analysis

Data were managed and analyzed in SPSS (Statistical Package for Social Sciences) version 23.^17^ A hierarchical block analysis using a Poisson model accommodated count outcome variables (*i.e.*, lifetime history of STIs). A logistic regression accommodated binary outcome variables (*i.e.*, concurrent partnerships). Predictor variables were self-reported current and life course experiences of trauma, stress, and violence. Each variable represented a construct measured by validated tools and/or subscales. Principal axis factoring with promax rotation determined the proxemic properties of these tools for this specific study sample. We disallowed cross-loading of factors and opted for items with greater loadings (>0.3) to measure the latent construct. Reliability analyses yielded high Cronbach's alphas (≥0.70) for all constructs.

#### CHT questionnaire

The CHT questionnaire is a validated tool that provides a self-reported rating of CHT.^18^ A 28-item short form of the original 70-item tool assessed emotional, physical, and sexual abuse. Participants were asked on a five-point Likert scale (1 = never true, 5 = very often true) whether when growing up they experienced abuse and neglect. Following removal of 3 validity items, the remaining 25 items distinctly loaded among 3 manifestations of CHT: (1) sexual abuse (5 items; range: 0–25), (2) physical abuse (9 items; range: 0–45), and (3) emotional abuse (12 items; range: 0–72). Of those, items that loaded together a CHT mean score for each dimension were developed. The greater the score, the more CHT that was endorsed. Three composite mean scores were developed: (1) sexual abuse, (2) physical abuse, and (3) emotional abuse.

#### Intimate partner violence

Experiences of IPV were assessed through self-reports of experiences of physical, emotional, verbal, or psychological abuse within a current intimate relationship. Questions used in this analysis were a subscale of four items (range: 0–20) from the HITS scale ^19^ that assessed the different dimensions of IPV (*i.e.*, How often does your partner physically hurt you?). An IPV mean was created to capture the occurrence of IPV currently being experienced, in which the greater the mean the greater the endorsement of these IPV experiences.

#### Lifetime stressor checklist-R

The life stressor checklist is a 30-item validated tool that assesses life events that are perceived to be frightening, upsetting, or stressful (*i.e.*, adult sexual harassment, robbery or physical assault, witnessing a murder).^20^ Participants reported whether these life events impacted their life at the time the stressful event occurred or if the event impacts them presently. Given the high correlation (Pearson = 0.828) between the two time frames, we opted to use a composite weighted mean score that distinctly measures the severity of the stressor at the time of the event (1 = not at all to 7 = extremely; range: 1–150). The greater the score, the greater the number and severity of the stressful events.

#### City stress inventory

The city stress inventory is an 18-item measure of experiences of neighborhood disorder and participant or family exposure to community violence.^21^ Neighborhood disorder measures self-reports from participants such as “how often was someone arrested or in jail” and violence exposure items such as “how often was a family member stabbed or shot?” The self-report inventory represented experiences from the preceding year using a five-point scale (0 = never to 3 = often, 8 = refuse to answer). The city stress inventory was measured with a neighborhood disorder factor inventory (7 items; range: 0–36) and a community violence factor inventory (11 items; range: 0–18).

#### Dependent variables

The dependent variables included in this analysis were selected based on the literature that demonstrates behavioral and contextual associations with HIV/STI acquisition among women in the United States. Limitations on the number of variables ensured their relevancy to populations who are disproportionally acquiring HIV. There are three dependent outcomes of interest in this current analysis: (1) *Lifetime history of sexual transmitted infection* is a count variable that measures the number of times women reported being treated for a sexually transmitted infection. (2) *Partner concurrency* is a dichotomized variable that indicates participants' reports of having had sexual relationships (primary or not) that overlapped in time within the past 3 months. (3) *Lifetime sex trading* is a count variable that indicates the number of sexual partners that are involved in an exchange of sex for survival-related resources (*i.e.*, food, money, drug use).

### Analysis

Standard data cleaning procedures were performed to ensure that data properties did not violate assumptions of the general linear model, and to assess the magnitude and mechanism of missing data.^22^ Participants who only had female partners (*n* = 5) were removed from the data set. Missing data were handled by either calculating a series mean for continuous variables, or setting the missing data to zero for dichotomized variables. To correct the skewness and normalize the distribution for the outcomes, lifetime STDs, the values were winsorized, and a log transformation was created for the outcome, lifetime sex trading. Listwise deletion was performed for each model eliminating cases that did not have complete data. Therefore, each model has a single *N*.

The predictors were added to the analysis in four phases based on the proximity of the life experience to the individual (interpersonal vs. neighborhood), the emergence of the experience across the life span (CHT vs. IPV), and the time frame of focus (lifetime vs. within the past year). The first level of analysis consisted of participants' demographics (age, race, family income, education, employment, history of being married vs. single). Level 2 comprised three separate dimensions of CHT (*i.e.*, emotional, physical, and sexual). Reports of lifetime experiences of IPV were entered in level three of the analysis. Level four, the full model, were reports of life stress that presently affected participants, neighborhood disorder, and family exposure to community violence. Three outcomes were regressed on each level: lifetime history of STI, lifetime sex trading, and current concurrency (sexual partners within the past 3 months).

A correlation matrix assessed the outcome variables association with one another and multicollinearity. The correlation analysis revealed that variables such as lifetime male partners and lifetime sex trading partners were correlated (Pearson = 0.732). We opted for lifetime sex trading partners due to its implication of an ecological vulnerability and that this outcome would intuitively influence reported number of male partners.

## Results

Many women reported at least one CHT experience and almost 70% of women reported lifetime experiences of IPV. Fewer women, however, reported IPV with their current partner (24%). Regarding life stress events, 30%–85% endorsed these experiences, ∼97% of women endorsed at least one or more forms of neighborhood violence, and up to 71% reported family exposure to community violence. Of the outcome variables assessed, ∼20% of women reported lifetime sex trading, 74.6% reported at least two STIs (mean = 2.8) in their lifetime, and 28% reported partner concurrency in the past 3 months. Bivariate analysis (Table 3) revealed that lifetime sex trading was the single dependent variable significantly associated with most predictors. The strongest associations with lifetime sex trading (*p* < 0.005) were emotional and physical CHT, lifetime stress, and neighborhood violence in the bivariate analysis.

**Table 3.** Bivariate Analysis Between Predictors and Outcome Variables

Outcomes	Lifetime sex trading		Concurrent partners		Lifetime STD/I	
Predictors	Pearson correlation	Significance	Pearson correlation	Significance	Pearson correlation	Significance
Age	0.150	0.014	−0.021	0.732	0.193	0.001
Race	−0.127	0.037	0.095	0.122	−0.151	0.013
Marital status	0.009	0.889	−0.061	0.320	−0.041	0.501
Employment	0.162	0.008	0.090	0.143	−0.017	0.745
Education	−0.148	0.015	−0.129	0.035	0.112	0.065
Family income	−0.175	0.004	0.066	0.281	−0.153	0.011
Childhood emotional trauma	0.240	0.000	0.097	0.113	0.072	0.237
Childhood physical trauma	0.250	0.000	0.116	0.057	0.137	0.024
Childhood sexual trauma	0.121	0.047	0.106	0.084	0.184	0.002
IPV	0.238	0.000	0.104	0.091	0.081	0.184
Life stress	0.287	0.000	−0.057	0.349	0.185	0.002
City violence exposure	0.122	0.046	0.062	0.309	0.111	0.067
Neighborhood disorder	0.195	0.001	0.051	0.404	0.137	0.023

In the hierarchical models (Tables 4–6), level one consisted of key demographics only. Across the different hierarchical regression levels, not one single demographic independent variable consistently predicted all three outcomes. Increased age and racial differences were predictors of lifetime history of STIs and lifetime sex trading at each hierarchical level. White women reported less STI history, but white and black women reported more lifetime history of sex trading than the other ethnoracial groups in this sample. Women who were ever married or currently married were less likely to report partner concurrency. Lower education predicted lifetime sex trading and concurrent partnerships in the past 3 months across the different levels. Lower family income was only associated with lifetime sex trading.

**Table 4.** Associations Between Reported Lifetime STI and Selected Covariates and Predictor Variables

N = 269	Block 1			Block 2			Block 3			Block 4		
	B	95% CI	Sig.	B	95% CI	Sig.	B	95% CI	Sig.	B	95% CI	Sig.
Age	0.021	0.012 to 0.030	0.000	0.019	0.010 to 0.028	0.000	0.019	0.010 to .028	0.000	0.018	0.009 to 0.028	0.000
Race: black	0.191	−0.18 to 0.401	0.074	0.229	0.018 to 0.440	0.034	0.230	0.019 to 0.442	0.033	0.237	0.025 to 0.448	0.029
Race: white	−0.499	−0.803 to −0.194	0.001	−0.422	−0.727 to −0.116	0.007	−0.420	−0.726 to −0.114	0.007	−0.388	−0.701 to −0.076	0.015
Marital status	−0.036	−0.278 to 0.207	0.772	−0.077	−0.320 to 0.165	0.532	−0.077	−0.320 to 0.166	0.534	−0.073	−0.318 to 0.172	0.560
Employment	0.028	−0.064 to 0.120	0.549	−0.008	−0.103 to 0.087	0.864	−0.009	−0.104 to 0.086	0.857	−0.011	−0.108 to 0.085	0.816
Education	−0.054	−0.137 to 0.029	0.199	−0.078	−0.161 to 0.005	0.067	−0.077	−0.160 to 0.007	0.073	−0.079	−0.164 to 0.005	0.066
Family income	−0.120	−0.240 to 0.001	0.052	−0.102	−0.223 to 0.020	0.102	−0.101	−0.223 to 0.021	0.105	−0.089	−0.214 to 0.036	0.162
Childhood emotional trauma				−0.044	−0.151 to 0.063	0.424	−0.044	−0.151 to 0.063	0.424	−0.046	−0.154 to 0.062	0.401
Childhood physical trauma				0.123	−0.017 to 0.263	0.085	0.120	−0.021 to 0.262	0.095	0.103	−0.043 to 0.249	0.166
Childhood sexual trauma				0.123	0.061 to 0.184	0.000	0.122	0.061 to 0.184	0.000	0.125	0.057 to 0.193	0.000
IPV							0.009	−0.212 to 0.231	0.936	−0.021	−0.251 to 0.210	0.861
Life stress										0.015	−0.058 to 0.088	0.680
City violence exposure										−0.029	−0.196 to 0.137	0.730
Neighborhood disorder										0.065	−0.042 to 0.171	0.234

CI, confidence interval.

**Table 5.** Associations Between Reported Number of Lifetime Sex Trading Events, Selected Covariates, and Predictor Variables

N = 265	Block 1			Block 2			Block 3			Block 4		
	B	95% CI	Sig.	B	95% CI	Sig.	B	95% CI	Sig.	B	95% CI	Sig.
Age	0.036	0.023 to 0.049	0.000	0.042	0.029 to 0.055	0.000	0.034	0.020 to 0.049	0.000	0.026	0.020 to 0.049	0.001
Race: black	0.908	0.2287 to 0.460	0.000	1.012	0.561 to 1.462	0.000	0.962	0.724 to 1.636	0.000	1.159	0.702 to 1.617	0.000
Race: white	0.673	0.124 to 1.221	0.016	0.802	0.255 to 1.350	0.004	0.796	0.246 to 1.346	0.005	0.891	0.321 to 1.461	0.002
Marital status	−0.390	0.219 to −0.820	0.076	−0.356	−0.789 to 0.078	0.108	−0.410	−0.854 to 0.034	0.070	−0.399	−0.857 to 0.058	0.087
Employment	0.238	0.085 to 0.391	0.002	0.114	−0.048 to 0.276	0.169	0.146	−0.017 to 0.309	0.080	0.106	−0.059 to 0.271	0.209
Education	−0.225	−0.357 to −0.094	0.001	−0.210	−0.342 to −0.078	0.002	−0.162	−0.290 to −0.034	0.013	−0.173	−0.303 to −0.042	0.009
Family income	−0.395	−0.619 to −0.170	0.001	−0.315	−0.545 to −0.085	0.007	−0.365	−0.596 to −0.134	0.002	−0.343	−0.585 to −0.101	0.005
Childhood emotional trauma				0.438	0.262 to 0.615	0.000	0.409	0.224 to 0.594	0.000	0.412	0.227 to 0.598	0.000
Childhood physical trauma				0.233	0.030 to 0.436	0.024	0.152	−0.061 to 0.364	0.162	0.019	−0.203 to 0.240	0.870
Childhood sexual trauma				0.071	−0.023 to 0.165	0.140	−0.018	−0.119 to 0.082	0.718	−0.060	−0.170 to 0.049	0.281
IPV							1.449	1.066 to 1.833	0.000	1.255	0.852 to 1.659	0.000
Life stress										0.192	0.095 to 0.290	0.000
City violence exposure										−0.111	−0.387 to 0.166	0.433
Neighborhood disorder										0.269	0.091 to 0.446	0.003

**Table 6.** Associations Between Reported Concurrent Partners over the Past 3 Months and Selected Covariates and Predictor Variables

N = 274	Block 1			Block 2			Block 3			Block 4		
	B	95% CI	Sig.	B	95% CI	Sig.	B	95% CI	Sig.	B	95% CI	Sig.
Age	−0.010	−0.022 to 0.002	0.108	−0.011	−0.023 to 0.002	0.089	−0.013	−0.026 to −0.764	0.049	−0.007	−0.020 to 0.006	0.291
Race: black	0.206	−0.438 to 0.026	0.082	0.174	−0.408 to 0.060	0.146	0.158	−0.393 to 0.077	0.187	0.113	−0.350 to 0.124	0.350
Race: white	−0.160	−0.124 to 0.443	0.270	−0.206	−0.079 to 0.492	0.156	−0.216	−0.071 to 0.503	0.140	−0.245	−0.049 to 0.540	0.103
Marital status	0.318	0.022 to 0.615	0.036	0.276	−0.023 to 0.576	0.070	0.282	−0.019 to 0.583	0.066	0.287	−0.017 to 0.591	0.065
Employment	0.078	−0.033 to 0.189	0.170	0.055	−0.059 to 0.169	0.345	0.059	−0.056 to 0.174	0.314	0.064	−0.054 to 0.183	0.287
Education	−0.156	−0.260 to −0.052	0.003	−0.166	−0.272 to −061	0.002	−0.160	−0.266 to −0.054	0.003	−0.139	−0.248 to 0.031	0.012
Family income	−0.012	−0.149 to 0.125	0.866	0.007	−0.132 to 0.146	0.923	0.010	−0.129 to 0.149	0.889	0.025	−0.117 to 0.166	0.732
Childhood emotional trauma				−0.021	−0.144 to 0.102	0.739	−0.029	−0.154 to 0.095	0.643	−0.011	−0.136 to 0.115	0.866
Childhood physical trauma				0.107	−0.058 to 0.273	0.203	0.094	−0.073 to 0.261	0.268	0.146	−0.024 to 0.316	0.092
Childhood sexual trauma				0.070	−0.007 to 0.147	0.073	0.060	−0.018 to 0.137	0.133	0.099	0.019 to 0.180	0.016
IPV							0.212	−0.049 to 0.474	0.112	0.291	0.120 to 0.561	0.035
Life stress										−0.171	−0.264 to −0.079	0.000
City violence exposure										0.091	−0.109 to 0.292	0.370
Neighborhood disorder										0.034	−0.099 to 0.167	0.618

For the second level, three dimensions of CHT were entered in the model: physical, emotional, and sexual. Physical CHT was not associated with any of the three outcomes on any level including the full model. Emotional CHT was implicated in lifetime sex trading, and sexual CHT was implicated in the lifetime history of STIs across the different levels including the full model. Sexual CHT was only significant for concurrent partnerships when IPV, life stress, and city stress were later added to the model. Lifetime IPV was added as a predictor in level 3 for each outcome and was positively associated with lifetime sex trading. In the full model, lifetime sex trading and concurrent partnerships were predicted by experiences of IPV.

In the full model, lifetime stress and city stress (neighborhood disorder and family exposure to community violence) were added. Lifetime stress was positively associated with lifetime sex trading and partner concurrency. Also in the full model, as neighborhood disorder increased, so did the number of lifetime sex trading partners. Family exposure to community violence was not associated with any of the outcomes. In the final model, there were no new predictors for any of the outcomes, however, being married no longer was associated with current partner concurrency.

## Discussion

Findings revealed that individual, interpersonal, and ecological factors posed distal and proximal threats to sexual health outcomes and behaviors among the women in this sample. The women in this sample reported extraordinary high levels of stress, trauma, and violence experiences across the life span. Various levels of CHT, IPV, life stress, and neighborhood violence were reported among over 50% of women in this study.

### Lifetime STI

The significance of lifetime STIs is it is often used as a sole proxy for adverse sexual health outcomes. Lifetime STIs are consistently associated with consequential HIV acquisition and influence lifelong sexual and reproductive health. In the final model, age, self-identified ethnoracial group (black vs. other designations), and childhood sexual trauma were associated with lifetime STIs. Increasing age provides greater opportunities to engage in sexual activity and may prolong one's exposure to STI acquisition. Dissolution of a long-term relationship in tandem with engagement in new sexual partnerships and less condom use is a source for increasing STI acquisition as one ages.^23,24^ Therefore, age as a predictor may have more to do with lifetime STI being an indication of lifetime sexual experiences rather than heightened STI incidence among women in this sample. In this sample, reports of a black racial identity predicted lifetime STI infection. Although racial designation is a demographic factor routinely explored as a predictor, research has established that experiences of social stigma and anti-black discrimination influence health outcomes such as STI acquisition among black persons.^25–27^

The associations between sexual CHT and sexual health outcomes and behaviors are well established.^28^ Early experiences of inappropriate and nonconsensual sexualization compromise one's physical, mental, and sexual health development. Specifically, sexual CHT is linked to adverse mental health outcomes, social dysfunction, and sexual compulsivity, which can manifest in behaviors such as early sexual debut, condomless sex with multiple partners, and other sexual behaviors that increase vulnerability to STI/HIV acquisition.^29–35^ Sexual CHT is consistently associated with sexual victimization and threats to sexual health in adulthood.^34^ One study among adult women who reported sexual CHT histories had a greater history of STIs and condomless sex.^16^ Findings in this study align with the literature in that sexual CHT showed a conclusive positive association with lifetime STIs. It has been demonstrated that women who experience sexual CHT are more likely to lack relationship power and control to engage in sexual health promotion with partners.^36^ Given the universal use of STI as a proxy for sexual health, research suggests that HIV/STI incidence results from a synergy of vulnerabilities, and perhaps is inadequate as a stand-alone indicator of adverse sexual health outcomes.^35^ This is particularly the case for those who have experienced IPV.^8^ Studies have also shown that among U.S. women, those who experienced IPV were more likely to report current or recurrent STIs, including HIV infection,^8,36^ and lack autonomy to promote sexual health (*i.e.*, condom use).^12^ In contrast to the literature, in this study, IPV was not associated with STI acquisition, but was a predictor for partner concurrency and sex trading.

### Current partner concurrency

The implications of partner concurrency among women is its consistent association with STI acquisition, including HIV infection.^37^ Partner concurrency among women has also been associated with early sexual debut, drug use during sex, and forced sex.^30^ Although an assessment of partner-based characteristics was beyond the scope of this analysis, to contextualize partner concurrency as a threat to sexual health, partner characteristics, relationship factors, and partner-based contexts are warranted to adequately characterize partner concurrency.^33,38,39^ For example, among women in this sample, ever having been or being currently married was no longer protective of partner concurrency once life stress and ecological factors were added into the full model. Likewise, for this sample, over half of the women reported ever experiencing IPV (70%). Current experiences of IPV (24%) were only predictive of partner concurrency when life stress and city stress, specifically neighborhood disorder, were added to the full model. Research has demonstrated that women in violent relationships have a heightened vulnerability to HIV/STI acquisition and condomless intercourse.^40^ In addition, although associations between concurrent partnerships and women's IPV experiences have been documented elsewhere, pathways and causation are not entirely understood.^41^ This suggests that the relationships among these experiences indicate a synergistic quality to the occurrence of partner concurrency. This would align with the literature on the syndemics of HIV/STI vulnerability and the experiences of violence and trauma.^42^ One study found that heterosexual women had a greater overall syndemic burden (substance use, victimization, and mental health) that was influenced more by stress than heterosexual men and men who have sex with men.^43^ Therefore, sexual health assessments in clinical and community settings require a holistic, comprehensive, and meaningful approach that captures partner-based information and behaviors to inform person-centered health promotion intervention.

Life stress events have demonstrated negative effects on sexual functioning and adverse mental health, but their effect on adverse sexual behavior is understudied.^44^ Research has demonstrated that although life stress events can result in poor sexual and relationship wellness and satisfaction,^44^ they have not been implicated in the influence on specific sexual behaviors and adverse sexual health outcomes among women.^44^ In the current study, women's experiences of lifetime stress were positively associated with partner concurrency, indicating that further investigation is warranted on the direct, indirect, or confounding influence of stress on threats to sexual health. Findings may suggest more synergistic relationships among these experiences.^45^ Qualitative investigations specifically could illuminate these associations on how women cope with life stress in the context of sexual health and behavior. Such investigation will inform intervention design and translation of findings in clinical and community settings. The significance of these findings also highlights the need for intervention that supports coping and resiliency for women who have experienced early childhood and life course stress and trauma and implicates enhancement of health and other service provider training to engage women in sexual health promotion.

### Lifetime sex trading

Sex trading can function as a service women provide to meet basic needs for daily living (*i.e.*, food and shelter) or may support substance use addiction.^43^ Research suggests that sex trading for goods, including those required for basic survival, may be a proxy of socioeconomic hardships and an indicator of lifetime poverty. In the full model, educational level and family income were both negatively associated with lifetime sex trading. Engagement in sex trading may also be an indication of unmet needs experienced by women who may lack the skills for gainful employment or who are underemployed, or who are experiencing a period of financial instability. Studies have found this was particularly the case for women who are living with HIV infection.^13,46^ This association demonstrates the interrelatedness of economic hardships and sexual health for women, while undergirding the need to focus on social determinants of health to effectively achieve sexual health promotion and mitigate vulnerabilities to adverse sexual health outcomes.^43^

As with partner concurrency, lifetime stress and IPV were positively associated with sex trading in this sample.^47,48^ Although sexual CHT predicted partner concurrency, emotional CHT was associated with lifetime sex trading in the final model. Specific manifestations of CHT (*i.e.*, sexual, emotional, and physical) can influence distinct outcomes along the life span and on sexual health outcomes. There is a dearth of literature that specifies the dimensions of CHT on sexual health outcomes and behaviors. However, sexual avoidance and compulsivity are hallmarks of CHT resulting in varying sexual experiences that may exacerbate adverse health outcomes.^47,48^ Our findings suggest that engagement in certain behaviors as adults may be indicators of experiences of particular dimensions of CHT that require distinct treatment modalities. Such experiences along the life course require certain prevention and treatment interventions for parents and families with children, and should assist adolescents and young adults to adopt therapies for healing from specific sources of trauma.

Certain health outcomes and engagement in behaviors manifest within an adversarial environmental context. In this sample, neighborhood disorder, and not family exposure to community violence, was positively associated with sex trading. Research has recognized the influences of the social and physical environments on the engagement in and experience of certain sex-based behaviors.^49–51^ Studies have cited ecological features of one's neighborhood as predictors of IPV experiences and consequential sexual health outcomes (*i.e.*, STI acquisition) among women.^52,53^ The physical (neighborhood) and social (criminal activity, social cohesion, over policing) environments can be sources of enduring stress, traumatic experiences, and enduring depravity that further threaten one's sexual health.^54,55^ Neighborhoods characterized as coercive sexual environments^53^ have a high acceptance of sexual harassment, sexual victimization, and sexual violence against women.^54,55^ This place-based phenomenon instills fear in girls and women due to blatant threats of sexual violence and interrupts healthy sexual development through early engagement in sexual activity and coerced sexual behaviors.^53^ This context and our findings implicate neighborhood quality and characteristics as antecedents and/or determinants of individual health behaviors and outcomes.

## Conclusion

Findings from this analysis have demonstrated the significance of the life course exposures to distinct forms of trauma, stress, and violence that uniquely and substantially contribute to morbidity among women.^8^ One underlying significance is the lasting effects of these experiences on impairment to cognitive functioning and disruption of neurobiological mechanisms,^56^ which may increase vulnerability to adverse sexual health outcomes.^47,57^

Clinical interventions for optimizing sexual health should include a thorough assessment of past and current dimensions of stress, trauma, and violent experiences. Services should address potentially maladaptive coping that may be resultant of these experiences and attempt to leverage resilient behaviors that can promote sexual health.^58^ Health providers' strategies should support self-healing and harnessing of personal assets to counter life course and ecological experiences of trauma, stress, and violence. A more holistic approach would also consider the role of environment and social trauma in IPV perpetuation to develop trauma-informed antiviolence intervention and policy.^59^

This analysis substantiates the importance of incorporating an ecological and life course perspective to investigate adverse sexual health outcomes among women. A focus on STI acquisition or partner concurrency in isolation of ecological factors is inadequate to understand and address women's experiences and vulnerability to adverse sexual health outcomes. In this sample, the predictive nature of CHT and IPV on sexual health behaviors and outcomes gained significance almost completely in the context of life and city stress, specifically neighborhood disorder. This relationship substantiates that sexual health-promoting interventions must include advocacy for safe environments, and a progressive social, educational, and economic policy. Such interventions can comprehensively address the interpersonal violence and poverty that disproportionately oppress women, and support policy that mitigates the economic burden that forces women to remain in violent relationships or engage in transactional sex. In addition, social policy, which decriminalizes commercial sex work, could potentially enable women to use personal sexual safety standards. Such decriminalization could increase condom use with transactional sex partners, and decrease STI/HIV acquisition.^60^

Due to the nature of this analysis, there are some limitations to the findings. The sample size is relatively small, but the reports of life events and experiences are representative of small urban centers. Relatedly, the proportion of women reporting adverse life events was high, however, the urban center overall suffers from high poverty and unemployment, which are represented in the sample. Certain key findings were not investigated but are pertinent for consideration in future studies. For example, mental health was not explored as a factor and it is unknown in this sample whether sex trading was exclusively an issue of financial instability. The literature on sex trading or transactional sex further requires a clearer delineation between transactions that may be rooted in a process of courtship or development of a romantic relationship and that which is coercive.^61^ The literature supports the need to examine partner characteristics, motivations, and partner behaviors that may heighten a woman's vulnerability to poor sexual health outcomes and engagement in behavior that fosters STI/HIV acquisition.^39^

## Abbreviations Used

CHT: childhood trauma
CI: confidence interval
IPV: intimate partner violence
STI: sexually transmitted infection
